# Effects of Increased CO_2_ on Fish Gill and Plasma Proteome

**DOI:** 10.1371/journal.pone.0102901

**Published:** 2014-07-24

**Authors:** Karine Bresolin de Souza, Fredrik Jutfelt, Peter Kling, Lars Förlin, Joachim Sturve

**Affiliations:** Department of Biological and Environmental Sciences, University of Gothenburg, Gothenburg, Sweden; University of California- Santa Barbara, United States of America

## Abstract

Ocean acidification and warming are both primarily caused by increased levels of atmospheric CO_2_, and marine organisms are exposed to these two stressors simultaneously. Although the effects of temperature on fish have been investigated over the last century, the long-term effects of moderate CO_2_ exposure and the combination of both stressors are almost entirely unknown. A proteomics approach was used to assess the adverse physiological and biochemical changes that may occur from the exposure to these two environmental stressors. We analysed gills and blood plasma of Atlantic halibut (*Hippoglossus hippoglossus*) exposed to temperatures of 12°C (control) and 18°C (impaired growth) in combination with control (400 µatm) or high-CO_2_ water (1000 µatm) for 14 weeks. The proteomic analysis was performed using two-dimensional gel electrophoresis (2DE) followed by Nanoflow LC-MS/MS using a LTQ-Orbitrap. The high-CO_2_ treatment induced the up-regulation of immune system-related proteins, as indicated by the up-regulation of the plasma proteins *complement component C3* and *fibrinogen β chain precursor* in both temperature treatments. Changes in gill proteome in the high-CO_2_ (18°C) group were mostly related to increased energy metabolism proteins (*ATP synthase*, *malate dehydrogenase*, *malate dehydrogenase thermostable*, and *fructose-1,6-bisphosphate aldolase*), possibly coupled to a higher energy demand. Gills from fish exposed to high-CO_2_ at both temperature treatments showed changes in proteins associated with increased cellular turnover and apoptosis signalling (*annexin 5*, *eukaryotic translation elongation factor 1γ, receptor for protein kinase C,* and *putative ribosomal protein S27*). This study indicates that moderate CO_2_-driven acidification, alone and combined with high temperature, can elicit biochemical changes that may affect fish health.

## Introduction

There is a scientific consensus that the oceans are becoming warmer and the water’s pH is decreasing [1]. As the atmospheric greenhouse gas levels rise, so do global mean temperatures, with an increment of 2–4.5°C predicted by the year 2099 [1], [2]. The Arctic is expected to experience the largest temperature changes, with increases of 4–7°C over land areas and 7–10°C over oceans [3]. In addition to the direct effects of high temperature, a temperature increase could also enhance the effects of other environmental stressors [4]. A known consequence of anthropogenic carbon dioxide (CO_2_) release into the atmosphere is the constant increase in dissolved CO_2_ in the oceans, which reduces the pH, a process known as ocean acidification (OA). Inevitably, marine organisms will be exposed to both OA and elevated temperatures simultaneously, with unknown physiological consequences.

Currently available data concerning the effects of high-CO_2_ in fish show significant changes in crucial physiological functions, such as growth, organ development, survival of larval fish [5], [6], and behaviour [7], [8]. Increased environmental CO_2_ levels result in higher physiological pCO_2_ [9], altering the acid-base physiology. In teleost fish, acidosis is prevented by active mechanisms, leading to chronically increased HCO_3_^−^ concentrations in blood plasma [9] and possibly reduced Cl^−^ and increased Na^+^ concentrations [10]. It has also been suggested that internal regulation is costly for fishes and could therefore cause a shift in energy expenditure away from fitness-enhancing processes such as growth or reproduction [11]. However, measurements of basal oxygen consumption in fish have failed to show this effect [9], [12].

Environmental proteomics is a growing field that investigates changes in the abundance and post-translational modifications of proteins. The link between genotype and phenotype is the proteome; thus, proteomics allows the discovery of biological processes behind physiological adaptations in response to environmental stressors. Proteomics is an approach for detection of sub-lethal shifts in protein expression, as many environmental challenges originate changes at the protein level [13], [14], before more pronounced biological modifications can be detected.

The aim of the present study was to identify the biological processes affected by OA in teleost fish at two different temperatures (optimal growth temperature, 12°C, and upper-tolerance temperature, 18°C) using proteomics. The fish chosen for this study was the Atlantic halibut (*Hippoglossus hippoglossus*), a semi-pelagic flatfish with large ecological and socioeconomical importance and widely distributed in cold waters of the North Atlantic Ocean.

## Materials and Methods

### Experimental animals, exposures, and water chemistry

All procedures regarding animal handling, exposure, and sampling were approved by the ethical committee of Gothenburg (ethical permits 221–2010 and 329–2010). The study was conducted at the Sven Lovén Centre for Marine Sciences, Kristineberg (Sweden), where Atlantic halibut from Fiskey’s hatchery station (Þorlákshöfn, Iceland) were used to perform the experiments. A total of 160 juvenile fish of both sexes, with an average weight of 16.4 g +/−0.2 g (SEM), were distributed into eight fish tanks with 100 L capacity (20 fish each). Each fish tank was supplied with aerated flow-through seawater at a rate of 2.5 L min^-1^ from its own header tank (200 L), and the header tanks were supplied with seawater pumped from a 32 m depth in the Gullmar fjord. The fish were kept at a 12∶12 h light:dark cycle and fed to satiation once a day, six times a week with commercial trout dry pellets (2 mm in size). The feed quantity provided was approximately 2.5% of the fish body mass per day.

The fish were exposed for 96 days to one of the four following treatments: 12°C, normal pH; 12°C, low pH; 18°C, normal pH; or 18°C, low pH. All treatments had duplicate tanks. The two temperatures were 12°C (11.71±0.03) and 18°C (17.88±0.05). Because Atlantic halibut is a cold-water species, 12°C is the optimal growth temperature for juveniles at high food availability, whereas 18°C is their upper-tolerance limit and results in a reduced growth rate [12], [15], [16]. The water temperature in the header tanks of the high-temperature treatment was increased from 12°C to 18°C over a period of 13 days, prior to the CO_2_ increase. The CO_2_ treatments were based on present day pCO_2_ 400 µatm (∼pH 8.0) and 1000 µatm (∼pH 7.6), representing the potential situation in the year 2100 in a fossil fuel-intensive scenario [1].

The water’s pCO_2_ was increased in the high-CO_2_ header tanks by CO_2_ bubbling, controlled by pH stat Computers (AB Aqua Medic, Bissendorf, Germany) connected to solenoid valves regulating the administration of pure CO_2_ into two header tanks at each temperature. The pH levels were manipulated only in the header tanks, thus reducing pH fluctuations in the fish tanks. The water parameters were monitored daily in both header tanks and fish tanks. Temperature and salinity were continuously recorded. The dissolved oxygen concentration (measured with a WTW Oxi 340i with an OxiCell 325 oxygen electrode, Weilheim, Germany) was kept at concentrations between 90–100%. Alkalinity was measured (with a Eppendorf BioPhotometer, Hamburg, Germany) according to Sarazin et al. [17]. The complete water carbonate chemistry was measured twice weekly using high-precision pH electrodes (WTW pH 3310 with a SenTix 41 electrode, Weilheim, Germany) and alkalinity measurements. pH_tot_ measurements were performed with TRIS-(2-amino-2-hydroxy-1,3-propanediol) and AMP (2-aminopyridine) [18]. The water carbonate chemistry data are presented in Table 1. Further details about the exposure procedures are available in Hernroth et al. [19] and Gräns et al. [12].

**Table 1:** Water parameters measured twice a week for 96 days in either sea water with the current pH or reduced pH (high-CO_2_).

Acclimation	CO_2_ Treatment	Replicate	Temperature	Alkalinity	pH_total_^in situ^	pH_total_ _scale_^STP^	pCO_2_^in situ^	pCO_2_^STP^	pCO_2_^N5^	pCO_2_^STP*N5^
Temperature (°C)			(°C)	(µmol kg^−1^)			(µatm)	(µatm)	(µatm)	(µatm)
12	Current pH	1	11.73±0.02	2244±51	7.99±0.01	8.19±0.01	468±11	262±6	243±6	373±6
		2	11.69±0.02	2266±34	7.99±0.01	8.18±0.01	478±13	268±8	249±7	379±7
	Reduced pH	1	11.71±0.02	2224±24	7.61±0.02	7.78±0.02	1223±52	727±34	636±27	964±27
		2	11.71±0.03	2234±26	7.57±0.02	7.74±0.02	1382±71	831±47	720±37	1048±37
18	Current pH	1	17.86±0.03	2226±27	7.87±0.01	8.16±0.01	642±14	276±6	184±4	381±4
		2	17.88±0.02	2170±26	7.86±0.01	8.15±0.01	643±15	276±6	183±4	381±4
	Reduced pH	1	17.86±0.07	2233±25	7.47±0.02	7.73±0.02	1791±85	854±45	513±25	1013±24
		2	17.90±0.06	2242±22	7.46±0.02	7.71±0.03	1861±105	892±58	532±31	1033±30

Data presented as means (±S.E.M) with two replicates per treatment. During the acclimation period, the salinity was 32.0±0.14 ‰, measured directly and simultaneously from the incoming sea water in all treatments. *^in situ^*indicates values at ambient temperature. ^STP^indicates values measured at standard temperature (0°C) and pressure (1 atm). ^N5^ indicates pCO_2_ values normalised to 5°C according to the isochemical temperature effect on seawater of 4.23%. ^STP*N5^indicates pCO_2_^N5^measured at standard temperature (0°C) and pressure (1 atm). Table modified from Gräns et al. (2014).

This experiment included several researchers from different fields, and the tissue obtained from the experimental fish was shared for use in distinct studies. For the present study, we collected samples from six fish individuals from each treatment (total of 24 fish). Fish were killed with a blow to the head. Gill filaments and blood plasma were sampled, flash frozen in liquid nitrogen, and stored at −80°C prior to analysis. Blood plasma was immediately extracted from the blood samples by centrifugation at 5000×g for 3 min. Since high-CO_2_ levels have been suggested to affect the physiology of marine fish through changes in acid-base regulation and gas exchange [9], [20], gills and blood plasma were chosen to be studied.

### Proteomic experimental design

The proteome of gills and blood plasma expressed under our experimental conditions was isolated in four different two-dimensional gel electrophoresis (2DE) runs. The resulting gels comprised four matchsets named Plasma 12°C, Plasma 18°C, Gills 12°C, and Gills 18°C. Each matchset contained six gels from control fish and six gels from fish exposed to high-CO_2_ (the Dodeca cell used to run the gels holds 12 gels per run). Every 2DE gel within the same matchset (same 2DE run) derived from different fish individual exposed to the same temperature (12°C or 18°C). The aim of the project was to focus on the effects of OA (combined or not with increased temperature); therefore, only gels belonging to the same matchset (same temperature) were compared. A between-run comparison of gels (from different 2DE runs) is not recommended in some cases, as it could lead to false positive results due to a series of reasons (such as differences between different cell types, tissues, and individuals, and methodical noise from the technique itself) [21], [22]. Hence, it was not the aim of the study to investigate the effects of temperature alone but only combined with the effects of high-CO_2_.

**Figure 1:**
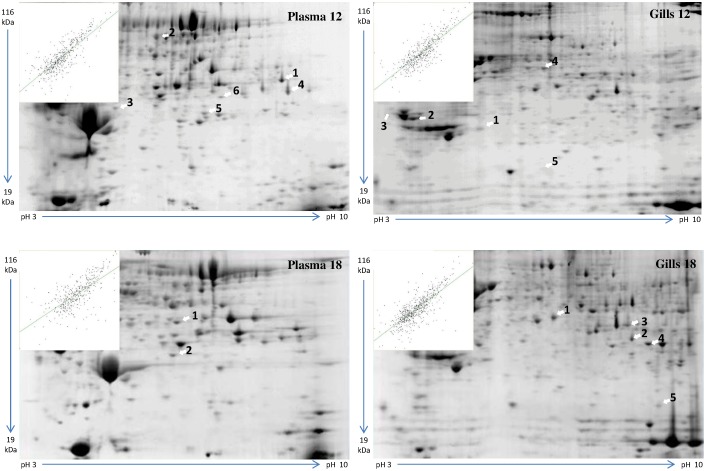
Representative images of the 2DE analyses. Each gel image represents one of the four 2DE runs and shows the position of the identified spots in plasma and gills (n = 12 per run, six controls and six exposed). The scatter plots show the relatedness of the replicate groups (control and exposed). The relative size (kDa) and *pI* of the regulated spots are also shown.

Data were divided per matchset and consisted of the protein spots identified (Figure 1). Because the main purpose was to screen major physiological changes in the fish, not all of the significantly regulated proteins were identified by MS/MS. The criteria used to choose the gel spots for identification were based on statistical significance and the positioning of the proteins in the gels. Proteins distant from each other were preferably selected to avoid the identification of the same protein isoforms in different spots.

### Two-dimensional gel electrophoresis (2DE)

2DE was carried out according to Albertsson et al. [23] using gill and blood plasma protein extracts. Prior to 2GE, the samples were purified with the Bio-Rad Protein Purification kit, a standard procedure to remove salts from marine samples. The blood plasma and gill samples were prepared in a buffer containing 40 mM Tris, 7 M urea, 2 M thiourea, 4% CHAPS, 0.2% (v/v) Biolytes, 100 mM DTT, 0.002% (v/v) BPB, endonuclease and 1 mM PMSF. Gills were homogenised in 250 µL of lysis buffer (referred to as sample buffer by Albertsson et al. [23]) for 4 sec using a VWR pellet mixer (VWR International - UK), followed by 4 sec of sonication. The homogenate was centrifuged for 20 min at 6500 rpm and then ultra-centrifuged at 4°C and 33,000×g for 1 h. The supernatant was collected and used for the 2DE analyses. Plasma samples were sonicated for 2 sec, and the homogenate was ultra-centrifuged at 4°C and 33,000×g for 1 h. Protein quantification of the supernatants was carried out using the RC-DC kit (Bio-Rad). For the first dimension, 100 µg of protein (200 µL) was applied to immobilised pH strips (11 cm, pH3-10 NL). The strips were actively rehydrated for 12 h at 50 V and 20°C. The isoelectric focusing was performed at 20°C in a Protean IEF cell according to a pre-programmed schedule: (1) 250 V for 1 h and (2) 8000 V until ∼35,000 V h was reached. The strips were equilibrated for 15 min in 50 mM Tris-HCl, 6 M urea, 30% (v/v) glycerol, 2% (w/v) SDS 2% DTT, and 0.002% (v/v) BPB. A second equilibration step was performed for 15 min with 2.5% iodoacetamide. Before the second dimension was carried out, the gel strips were soaked in electrophoresis buffer (25 mM Tris; 192 mM glycine; 0.1% SDS). Electrophoresis was carried out in a Criterion Dodeca Cell using a Power Supply Model 200/2.0. The gels were run through the stack for 20 min at 50 V and then for ∼1 h at 200 V (constant voltage). Thereafter, the gels were stained with BIO-SAFE Coomassie according to the manufacturer’s instructions.

### Scanning and computer-assisted gel analysis

The resulting gels were scanned using an Epson Expression 1680 Pro scanner in professional mode. The digitalised images at 16-bit grey scale and 400 dpi were saved in TIFF format. Gel image comparisons were performed using PDQ 7.3.0 (Bio- Rad).

### Protein digestion and peptide extraction

Gel spots found to be significantly affected by the treatments were excised and digested with trypsin according to Shevchenko et al. [24], with minor modifications [25]. The gel pieces were de-stained by washing three times with 25 mM NH_4_HCO_3_ in 50% CH_3_CN and then dried in a vacuum centrifuge and incubated with 10 µL of digestion buffer (50 mM NH_4_HCO_3_, 10 ng/µL trypsin) at 37°C overnight. The digestion process was stopped, and the extraction was initiated by adding 10 µL of 2% CF_3_COOH/75% CH_3_CN. The peptides were further extracted in 50% CH_3_CN/0.2% CF_3_COOH, and the supernatant was evaporated in a vacuum centrifuge. The peptides were re-suspended in 0.1% HCOOH and transferred to glass vials prior to the MS/MS analysis.

### Peptide identification with mass spectrometry

Nanoflow analysis was performed at the Proteomics Core Facility of University of Gothenburg. Injections of 2 µL of the samples were analysed with an HTC-PAL autosampler (CTC Analytics AG, Zwingen, Switzerland) connected to an Agilent 1200 binary pump (Agilent Technologies, Palo Alto, CA, USA). The peptides were trapped on a pre-column (45×0.075 mm i.d.) and separated on a reversed-phase column (200×0.050 mm). Both columns were packed in-house with 3 µm Reprosil-Pur C_18_-AQ particles. The analytical column flow through was reduced with a split of approximately 100 nL/min. For liquid chromatography (LC), a 25-min gradient of 5–35% CH_3_CN in 0.2% HCOOH was used to isolate the peptides. The mass spectrometry (MS) analysis was performed using a LTQ-Orbitrap XL (Thermo Scientific) with the following settings: spray voltage of 1.4 kV and MS1 scans at 60,000 resolution (*m*/*z* 400) with full MS mass range of *m*/*z* 400–2000. The LTQ-Orbitrap was operated in data-dependent mode with CID (collision induced dissociation) fragmentation of the six most intense double- or triple-protonated ions from each MS1 precursor ion scan.

### Database search

All tandem mass spectra were analysed using Thermo Proteome Discoverer software (v. 1.2, Thermo Scientific) with the Mascot search engine (v. 2.3.2 Matrix Science, London, UK) and the NCBI database version April 2012. The search parameters were set to bony vertebrates, MS accuracy of 5 ppm, MS/MS accuracy of 0.5 Da (for fragmentation in the linear ion trap with CID), one missed cleavage by trypsin allowed, dynamic carbamidomethyl modification of cysteine (previous tests show that this modification is not 100%), and oxidised methionine. For protein identification, the minimum criteria were two tryptic peptides matched at or above the 99% confidence level, allowing only rank-one peptides and peptides in top-score proteins. No decoy database was used, but the highest confidence level for Mascot significance threshold together with two or several peptides for one protein that can ensure the identification.

### Statistics

The nonparametric Mann-Whitney U-rank test was used to analyse the intensities of the matching spots from different gels of the same matchset to verify the differences in gill and blood plasma protein expression between fish exposed to high-CO_2_ compared to the controls (from the same temperature treatment). The protein intensity of each spot was normalised to the total intensity in each gel image. The data and number of spots/gel are reported as means ± SD. The alpha level was set to a *p*-value ≤0.05 in all the analyses.

## Results

A summary of the 2DE findings is displayed in Table 2, showing the number of spots detected, total number of spots regulated, and number of identified spots (chosen to be analysed by MS/MS) in every 2DE run. The Gills 18°C matchset has the highest number of detected spots as well as the highest standard deviation (SD). The matchset Plasma 12°C has the highest number of spots that were significantly affected by the CO_2_ treatment.

**Table 2:** Summary of the 2DE analyses carried out in plasma and liver of Atlantic halibut.

Matchset	Number of spots detected (mean ± SD)	Spots significantly different from controls	Spots identified
**Gills 12°C**	452±58	6	5
**Gills 18°C**	611±92	10	5
**Plasma 12°C**	455±67	26	6
**Plasma 18°C**	398±85	4	2

Every matchset represents the results obtained in a distinct 2DE run. The effects of high-CO_2_ were tested within gels belonging to the same matchset (n = 12, six controls and six exposed). Mann-Whitney U-rank test and *p*-value ≤0.05.

The master images from each of the 2DE runs showing the location of each identified spot are shown in Figure 1, together with a respective scatter plot showing the relatedness of the replicate groups (control and exposed). The proteins identified in each excised gel spot are shown in Table 3. The statistical significance, regulation, protein name, species, proposed function, number of peptides, accession number, and score are listed for every spot. The complete protein identification data is available as Table S1, including the specific peptide sequences for each protein.

**Table 3:** Regulated proteins identified by MS/MS.

Matchset	Spot	*p*-value	Regulation	Protein Name	Species	Proposed Function	Pep.	Accession	Score
**Gill 12 °C**	1	0.002	Up	Annexin 5	*Anoplopoma fimbria*	Apoptosis Signalling	6	229366222	569
				Annexin max1	*Epinephelus bruneus*	Apoptosis Signalling	2	328677117	337
				Annexin 5B	*Danio rerio*	Apoptosis Signalling	2	160773369	130
	2	0.005	Down	Cytoskeletal tropomyosin	*Coturnix coturnix*	Cellular Structure and Motility	14	833603	3011
				Tropomyosin 4-α	*Takifugu rubripes*	Cellular Structure and Motility	11	28557136	1686
				Tropomyosin 4-α	*Salmo salar*	Cellular Structure and Motility	11	213515262	1575
	3	0.041	Down	Glyceraldehyde 3-phosphate dehydrogenase	*Acanthopagrus schlegelii*	Multifunctional/Energy Metabolism	2	89147695	129
	4	0.022	Up	Enolase 1- α	*Salmo trutta*	Multifunctional/Energy Metabolism	8	11999265	1061
				Enolase 1- α	*Gillichthys mirabilis*	Multifunctional/Energy Metabolism	9	226441951	775
				Enolase- α	*Acipenser baerii*	Multifunctional/Energy Metabolism	10	98979415	773
	5	0.023	High-CO_2_	Eukaryotic translation elongation factor 1γ	*Pseudopleuronectes americanus*	Protein Biosynthesis/Apoptosis Related	3	28394501	150
**Gill 18 °C**	1	0.047	Up	Fructose-1,6-bisphosphate aldolase	*Sphoeroides nephelus*	Energy Metabolism	6	2828145	1698
	2	0.007	Up	Receptor for activated protein kinase C	*Oreochromis mossambicus*	Inflammation Mediator/Apoptosis Related	9	37498964	1844
	3	0.038	Up	Malate dehydrogenase 1A	*Danio rerio*	Energy Metabolism	7	41053939	1356
				Malate dehydrogenase thermostable	*Sphyraena idiastes*	Energy Metabolism	8	14583129	1169
	4	0.041	Up	ATP synthase subunit α	*Salmo salar*	Energy Metabolism	6	209151440	505
	5	0.046	Up	Putative ribosomal protein S27	*Oncorhynchus mykiss*	Protein Biosynthesis/Apoptosis Signalling	2	14787421	79
**Plasma 12°C**	1	0.049	Up	Fibrinogen β chain precursor	*Larimichthys crocea*	Inflammation Regulator/ImmuneSystem Related	4	218665023	384
				Fibrinogen β chain precursor	*Paralichthys olivaceus*	Inflammation Regulator/ImmuneSystem Related	3	146447341	213
	2	0.041	Down	IgM heavy chain constant region	*Hippoglossus hippoglossus*	Immune Response Mediator	8	7769631	804
	3	0.009	High-CO_2_	Apolipoprotein AI precursor	*Platichthys flesus*	Immune System Effector/Lipid Carrier	4	60417202	686
	4	0.043	Down	Module-substituted chimera haemoglobin β-α	*–*	–	6	4929993	395
	5	0.048	High-CO_2_	Complement component C3	*Paralichthys olivaceus*	Immune System Effector	8	6682835	365
	6	0.045	Up	Complement component C3	*Dicentrarchus labrax*	Immune System Effector	4	339269297	481
**Plasma 18°C**	1	0.040	Up	Fibrinogen β chain precursor	*Larimichthys crocea*	Inflammation Regulator/ImmuneSystem Related	6	218665023	1228
	2	0.008	Up	Complement component C3	*Hippoglossus hippoglossus*	Immune System Effector	9	58373439	627

The effects of high-CO_2_ exposure were studied at two temperatures (12°C and 18°C) (n = 12, six controls and six exposed). Spots classified as “up” regulated are those containing increased amounts of protein; spots “down” regulated are those with reduced amounts of protein; spots classified as “high-CO_2_” are those detected only in the high-CO_2_ group (but not in the control group). The accession number is an identifier given to the protein according to the NCBI database, and the score is the sum of the unique ion scores. “Pep.” represents number of peptides. Complete protein identification data is available as Table S1, including the specific peptide sequences for each protein. Mann-Whitney U-rank test and *p*-value ≤0.05.

In matchset Gills 12°C, two spots were up-regulated (*annexin* and *enolase-α*) and two down-regulated (*tropomyosin* and *glyceraldehyde 3-phosphate dehydrogenase*); and one protein was identified by the software only in the high-CO_2_ group (*eucaryotic translation elongation factor 1γ*). Proteins identified in only one of the groups (control or high-CO_2_) could actually be present in both groups but in low, non-detectable amounts. Spots 1, 2, and 4 display different isoforms of the same protein within the same spot.

In matchset Gills 18°C, all five identified proteins were up-regulated in the high-CO_2_ exposed group (*fructose-1,6-bisphosphate aldolase, receptor for activated protein kinase C, malate dehydrogenase thermostable* and *malate dehydrogenase 1A, ATP synthase subunit α*, and *putative ribosomal protein S27*). Spot 3 shows two isoforms of *malate dehydrogenase*, one of which is thermostable. Spots 1, 2, 4, and 5 have only one protein isoform.

The matchset Plasma 12°C has two up-regulated spots *(fibrinogen β chain precursor* and *complement component C3*), two down-regulated spots (*IgM heavy chain constant region* and *module-substituted chimera haemoglobin β-α*), and two spots that were only identified in the high-CO_2_ group (*apolipoprotein AI precursor* and *complement component C3*). Spot 1 contains two isoforms of the same protein, and spots 5 and 6 also consist of different isoforms of the same protein (*complement component C3*).

**Figure 2:**
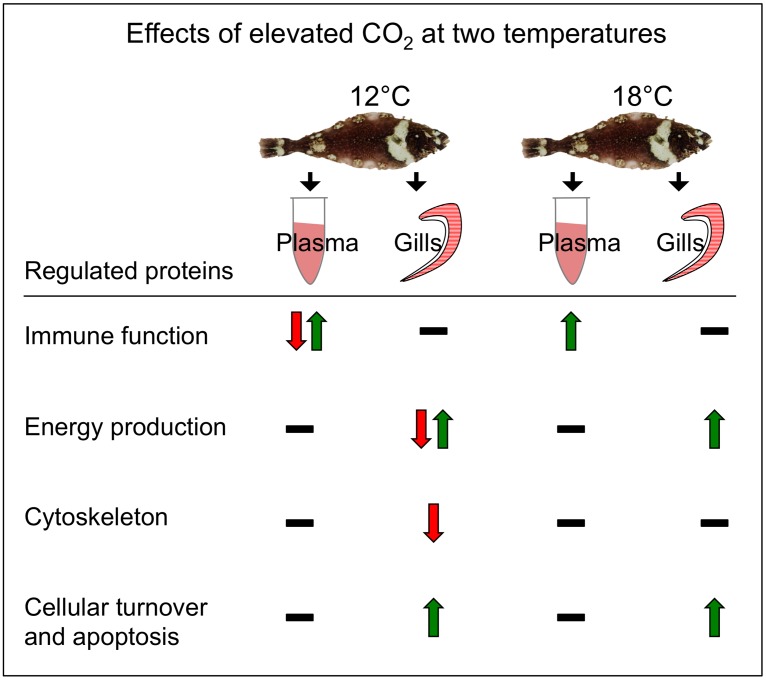
Study summary and main findings. We analysed gills and blood plasma of Atlantic halibut (*Hippoglossus hippoglossus*) exposed to temperatures of 12°C (control) and 18°C (impaired growth) in combination with control (400 µatm) or high-CO_2_ water (1000 µatm) for 14 weeks. The proteome analysis was performed using (2DE) followed by Nanoflow LC-MS/MS. The main systems affected are listed. Green arrows represent up-regulation, red arrows represent down-regulation, and black dashes represent no protein regulation.

In matchset Plasma 18°C, only two spots were identified. Spot 1 has the same protein found in spot 1 from matchset Plasma 12°C (*fibrinogen β chain precursor*), whereas spot 2 (Plasma 18°C) has the same protein (*complement component C3*) found in spots 5 and 6 of matchset Plasma 12°C. However, all three spots consisted of different isoforms of the same protein. The results from matchset Plasma 18°C indicate that high-CO_2_ alone can induce the up-regulation of *fibrinogen β chain precursor* and *complement component C3* in blood plasma, as they were found up-regulated in both temperature treatments. In our study, most peptide matches consisted of homologous counterparts in other vertebrates (mostly teleost fish, Table 3), as the complete halibut genome is not yet published. Spots displaying different isoforms of the same protein are a result of the LC-MS/MS accuracy and reflect the molecular diversity of the cells [26]. A general overview of the study and the main findings are summarised in Figure 2.

## Discussion

Proteomics is a hypothesis-generating method, used here to provide a holistic view of the physiological effects of high-CO_2_ in teleost fish and to lay a foundation for future studies. To our knowledge, this is the first study addressing the effects of OA on fish proteome.

### Increased expression of energy metabolism proteins

An organism’s ability to cope with environmental stress can be evaluated by investigating physiological conditions, especially energy status, as the energy supply can be a limiting factor for physiological processes. During environmental challenges, additional energy is required to supply numerous metabolic processes in response to such environmental conditions [27]. Among our findings in gills, energy-generating enzymes are up-regulated in the high-CO_2_/high-temperature (18°C) group. The up-regulation of such enzymes (*ATP synthase, malate dehydrogenase, malate dehydrogenase thermostable*, and *fructose-1,6-bisphosphate aldolase*) is possibly coupled to a higher energy demand and/or increased protein synthesis [28], [29], therefore indicating changes in the energetic balance between energy expenditure and requirements. The need for ionic and pH regulation under low pH conditions could increase energetic needs, which is in accordance with experiments in Atlantic cod (*Gadus morhua*), whereby the maximal Na^+^/K^+^-ATPase activity was increased in the gills of high-CO_2_-exposed fish [9]. These indications of increased energy demand suggest that OA has a metabolic cost for fish.

Gills exposed to high-CO_2_ at control temperature (12°C) also show modifications in proteins related to energy generation. In addition to their glycolytic role, *enolase-α* (up-regulated) and *glyceraldehyde 3-phosphate dehydrogenase (GAPDH)* (down-regulated) are multifunctional proteins with multiple catalytic functions. *Enolase-α* can provide cellular protection by increasing anaerobic metabolism [30], whereas *GAPDH* is described as DNA-protective [31] and involved in apoptosis [32]. Energy shifts have also been reported previously in gills of marine fish exposed to high-CO_2_ [28], [29], with the energy requirements changing to supply the cells with enough energy for ion and acid-base regulation.

Previous proteomic studies in aquatic organisms show that environmental pollutants can modify the abundance of energy metabolism proteins. The proteome of crab gills (*Eriocheir sinensis*) exposed to heavy metals showed changes in the abundance of several proteins involved in energy metabolism, such as *GAPDH, ATP synthase*, and *malate dehydrogenase* [33], also regulated in the present study. Related findings are shown in a proteomic study on fish (*Pimephales promelas*) exposed to 17β-trenbolone (androgenic) and flutamide (antiandrogenic), where both chemicals altered the hepatic abundance of ATP-production enzymes, such as *GAPDH, ATP synthase*, and *fructose-1,6-bisphosphate aldolase* [34].

### Indications of increased cellular turnover and apoptosis

Increased cellular turnover is a process associated with cell death, division, and/or differentiation. A higher rate of cell turnover in mucosal/epithelial tissue can improve the maintenance of the protective barrier against environmental insults, but apoptosis can also signal stress and epithelial disturbances [35], [36]. Such changes in gill epithelium were reported in fish after the exposure to environmental pollutants [37] and occurred in combination with increased influx of leucocytes to the gills [38] as part of the defence mechanism. Our findings in gills suggest increased cellular turnover, as supported by the up-regulation of proteins (two proteins from each temperature treatment) involved in apoptosis and cell proliferation. Here, the increased expression of *annexin (5 and max 1)* and *eukaryotic translation elongation factor 1γ (EF1γ)* in the high-CO_2_ (12°C) group, together with the up-regulation of *receptor for activated protein kinase C* and *putative ribosomal protein S27* in the high-CO_2_ (18°C) group suggest that high-CO_2_ can induce an increase in cell proliferation and apoptosis in gill cells. *Annexin 5* is a widely used biomarker for apoptosis [39], and *EF1γ* and *putative ribosomal protein S27* also play a role in apoptosis and protein synthesis [40], [41]. The *protein kinase C (PKC)* family is linked to cellular turnover [42], and mediates inflammation [43] and apoptotic cell death [44]. Therefore, the up-regulation of the *receptor for protein kinase C* found in the high-CO_2_ (18°C) group could be another indication of higher cell turnover/apoptosis. However, no specific *PKC* isoform was found regulated in any treatment, which restricts further conclusions about the biological processes possibly affected.

Fish gills normally have a high rate of protein turnover compared to other tissues [45]. However, increased cellular turnover is regarded as part of the stress response in aquatic organisms [46] and has previously been observed in the gills of mussels exposed to polluted water [47] and in freshwater fish exposed to acidified water [48]. In addition, a study with the fresh water fish Mozambique tilapia (*Oreochromis mossambicus*) shows an increase in cellular turnover associated with higher amounts of mitochondria-rich cells (chloride cells) in gills when the fish were exposed to acidified waters [46]. The same study shows that chloride cells turn over quickly in acidic water, and the number of apoptotic and immature cells also increased markedly with the exposure to acidification. Deigweiher et al. [29] reported that hypercapnia induced increases in protein and RNA synthesis in gills of two Antarctic fish species (*G. gibberifrons* and *N. coriiceps*). These findings were also coupled to a high energy demand due to higher ionic exchange, which is in accordance with the outcomes of the present study.

The preservation of acid-base stability as well as ionic and osmoregulatory homeostasis require physiological modifications to sustain fish acclimation to future CO_2_ levels. These modifications involve acid-base compensation mechanisms and consequent ionic exchange with the environment [10]. Such compensatory mechanisms include the rearrangement of structural proteins as *tropomyosins*, which are related to intracellular transport and cellular secretion, motility, and structure [49]. Three different isoforms of *tropomyosin*, found here to be down-regulated in gills (12°C), compose structural and focal adhesion units. Several studies have related the down-regulation of *tropomyosins* with the loss of normal structure in disturbed cells [50], as a reduced expression of *tropomyosins* is often related to cellular membrane disturbances, a typical effect in gills with increased ionic exchange. Similar effects were reported in a study on clams (*Chamaelea gallina*) exposed to copper, PCBs, tributyltin, and arsenic [14]. That study also showed changes in several cytoskeletal proteins (including *tropomyosin*), suggesting that environmental pollutants can potentially change the cytoskeleton of cells and affect cellular conformation.

### Changes in immune system-related proteins

Fish rely on the innate immune system for protection against potentially harmful conditions [38], [51]. Recent studies show that the immune system is one of the biological systems affected by pollutants in fish [52], and such immune responses can be assessed by measuring specific plasma proteins [53]. A proteomics-based study on juvenile cod (*Gadus morhua*) exposed to oil and oil spiked with alkylphenols/PAH also showed changes in the composition of plasma proteins [54], indicating effects on fibrinolysis, the complement cascade, the immune system, fatty acid metabolism, and increased proteins associated with apoptosis. In our study, we found increased expression of different isoforms of *complement component C3 (CC3)* and *fibrinogen β-chain precursor* in the plasma of both high-CO_2_-exposed groups (12°C and 18°C), suggesting that the trigger was high-CO_2_ alone, independent of temperature (Table 2). *CC3* plays a central role in the innate immune system, supporting the activation of all three pathways of the complement system in teleost fish: the classical, alternative, and lectin pathways [55]. Fibrinogen expression regulates inflammation in several tissues, and a high serum fibrinogen level is a biomarker for a pro-inflammatory state [56]. In addition, the down-regulation of *IgM heavy chain constant region* (high-CO_2_ 12°C group) is consistent with these findings, since it mediates immune responses and is reported to be up-regulated in early stages of immune responses in fish [57]. The down-regulation of this component could be a consequence of the long term high-CO_2_ exposure, given that the immune system changes the form of response over time [53].

Environmental changes can activate physiological modifications, providing cells with means to cope with challenges. Therefore, the up-regulation of *apolipoprotein AI precursor*, identified here only in the high-CO_2_ (12°C) group, might be related to a high-energy turnover once *apolipoprotein AI* functions as a lipid carrier in the blood [58]. In addition to its lipid transport role, *apolipoprotein AI* has antimicrobial and antibacterial functions in fish [59], [60], and it is an important effector of innate immunity [61].

Recent studies have demonstrated the effects of high-CO_2_ exposure on the immune system of bivalves [62], echinoderms [63], and crustaceans [19]. The results of our study indicate that high-CO_2_ induced immune activation in teleost fish, which is the opposite effect found in invertebrates. The difference may be due to a poor pH regulation in the extracellular fluid of invertebrates, whereas fish maintain an appropriate pH but increase their plasma HCO_3_. To our knowledge, this is the first example of changes in the immune system of fish subjected to OA experiments.

## Conclusions

Understanding the capacity of teleost fish to acclimate and possibly adapt to future environmentally relevant CO_2_ concentrations is critical in order to predict the biological impacts of OA. This study provides insights concerning how teleost fish are affected by high-CO_2_. We show changes in the regulation of proteins that suggest immune system stimulation, increased cell apoptosis and turnover in gills, and increased gill energy production. The results presented here support the hypothesis that the CO_2_ levels estimated to occur at the end of this century will pose physiological challenges to fish, and the consequences of these challenges are widely unknown.

## Supporting Information

Table S1 **Protein identifications with sequence data.** Data exported from Thermo Proteome Discoverer software using the Mascot search engine and NCBI database. The information provided for every protein includes coverage, peptide-spectrum matches (PSMs), number of peptide sequences, molecular weight, total score, peptide sequence, modifications, ion score, expected value, threshold for significant match at 99%, homology threshold, peptide mass and charge, retention time and spectra information.
(XLSX)
